# The Role of Discrete Emotions in Risk Perception and Policy Support during Public Health Crises: The Moderation Effect of SNS Dependency

**DOI:** 10.3390/ijerph182111654

**Published:** 2021-11-06

**Authors:** Soohee Kim

**Affiliations:** College of Communication, Yonsei University, Seoul 03744, Korea; soohkim@yonsei.ac.kr

**Keywords:** discrete emotions, risk perception, policy support, social media, SNS dependency

## Abstract

People often experience strong emotions during public health crises. This study examines how these emotions shape the perception of risk and support for policies to address the risk. In particular, this study explores the moderating effects of social network services (SNS) use in the process, considering that SNS have become a prominent communication platform during crises. Using a nationwide online survey conducted on the issue of fine dust air pollution in South Korea, this study found that feelings of anxiety, anger, and sadness about the risk issue were positively related to risk perception and policy support, while the relative effects of these emotions varied. Furthermore, the results demonstrated that reliance on SNS for learning (i.e., SNS learning dependency) moderated the influence of anxiety: the positive impact of anxiety was greater among those who used SNS for learning, while no such effects were found among those who used SNS for play or entertainment (i.e., SNS entertainment dependency). The implications of this study are discussed in terms of the distinct role emotions play in public responses to risks and the importance of considering the impact of SNS on public perceptions and judgments in this era of social media.

## 1. Introduction

The role of emotion in public risk perceptions and judgments has received increasing attention among scholars. While early studies primarily focused on cognitive mechanisms to explain how people form perceptions and judgments about risks, research has increasingly documented the important role that emotion plays in the context of risks [1,2]. For example, guided by cognitive appraisal theories of emotion, scholars have emphasized that discrete emotions, associated with distinct appraisals of situations, exert a powerful influence on subsequent judgment and decision-making in various contexts of risks [3]. Evidence showed that specific emotional responses, rather than the systematic processing of information, determine perceptions and critical decisions about risks [4].

At the same time, researchers have consistently documented that the impact of discrete emotions can be largely influenced by the way individuals use media [5,6]. This research suggests that how individuals use certain media—or specific genres of media—can change the way individuals interpret given issues or situations, thus, triggering distinct emotional responses. In particular, recent research has demonstrated that social network services (SNS), as an emerging platform for communication in risk contexts, plays distinct roles in shaping emotional responses about given risks [7,8]. That is, SNS use can exert powerful influence on risk-related judgments because it can influence the extent to which specific emotions are triggered in response to certain risk issues.

While this line of research together identifies the possibility that distinct patterns of SNS use differentially influence the experience of specific emotions or the strength of these emotions, research has yet to systematically examine how specific types of SNS use influence the relationship between discrete emotions and risk judgments. This study aims to fill this gap by exploring how reliance on SNS for specific goals (i.e., learning or entertainment) may exert distinct influences on the overall impact of discrete emotions in the context of public health risk [9].

Therefore, the present study aims to address two questions. First, this study examines how discrete emotions shape risk perceptions and judgments during public health risk. Second, this study investigates the potential moderating effects of specific types of SNS use (i.e., learning and entertainment) in the relationship between emotions and risk-related judgments. By taking a closer look at how specific SNS uses may influence the impact of discrete emotions, this study seeks to better understand the impact of discrete emotions in the current media environment, with the context that social media has become a prominent platform for risk communication.

To the best of our knowledge, this study is the first to examine the interactions between discrete emotions, specific SNS uses, and risk judgments. To examine these questions, this study used an original online survey data from a national sample of South Korean adults in the context of fine dust air pollution. As a newly emerging environmental concern, fine dust air pollution has been increasingly recognized as a major public health concern [10] as this problem has been significantly correlated with increased human mortality and long-term effects, such as lung cancer and myocardial infarction [11].

The fine dust problem has been a social issue in Korea since 2013 [12], and the problem has worsened in recent years. For example, in 2019, the worst levels of fine dust pollution were observed throughout the country [13]. Accordingly, news media has paid extensive attention to the issue of fine dust air pollution, while this news coverage of fine dust has been consistently criticized for their emotional coverage of the issue [14]. Furthermore, as an environmental risk, the issue of fine dust pollution has been characterized as one that contains high uncertainty and the sense of situational controllability, which were shown to be the two central determinants of risk judgments [2]. Thus, this study aims to contribute to a better understanding of the underlying mechanism of how discrete emotions shape public responses to risk in this era of social media.

In the following, I first discuss the literature on discrete emotions and the relationship with risk-related perceptions and judgments. In doing so, the present study particularly focus on three discrete emotions—anxiety, anger, and sadness—because these emotions are shown most frequently triggered when individuals encounter certain threats related to their health, including public environmental or health crisis [15,16].

## 2. Literature Review

### 2.1. Discrete Emotion, Risk Perception, and Judgment

Before discussing the role of emotion in risk perception and judgment, it is worth briefly mentioning the conceptual difference between risk and uncertainty, as they are closely related but distinct concepts. As intertwined concepts in a risk experience [17], risk and uncertainty have been conceptualized as known and unknown uncertainties [18]. Specifically, risk is considered a situation where the probabilities or outcomes of an action are known, whereas uncertainty is considered as a situation under which such information is not available [18]. Therefore, appraisals of uncertainty are linked to perceptions of risk [19]. In fact, the research on risk communication has examined uncertainty as a core aspect of cognitive appraisal of the situation, which can affect risk-related judgment or behavior.

The literature on risk perception and judgments has increasingly emphasized that emotion plays a critical role in perceptions and judgments about risks. Although research has long emphasized the role of cognitive processes to understand how individuals make judgments and engage in preventive behaviors in the face of crises, recent research has increasingly incorporated discrete emotions as a prominent predictor of risk perceptions and judgments. Various theories of emotion suggest that emotions can change the way individuals perceive and make judgments about risks.

For example, the “affect as information” hypothesis [20] proposes that affective states serve as a heuristic cue that guides subsequent judgment and decision making. According to this hypothesis, affect can play a primary role in motivating judgment and behavior since reliance on affective states is easier and quicker to make judgments about complex and uncertain situations. While this affect heuristic approach provides a useful framework to understand aspects of risk perception, scholars have argued that this approach is limited as it cannot explain the distinct effects of discrete emotions belonging to the same negative or positive affect. For example, research showed that fear and anger, while both belonging to negative affect, had differential influences on perceptions and behavioral judgements regarding a risk [21].

Accordingly, scholars have proposed the importance of considering the distinct characteristics of specific emotions. For instance, research on appraisal tendency framework (ATF) [22] has suggested that discrete emotions are associated with distinct appraisal dimensions, such as responsibility, certainty, control, pleasantness, anticipated effort, and attention [23]. According to this view, each emotion is associated with specific judgments about situations, and thus each emotion triggers distinct judgments and behavioral intentions. For example, anger is associated with high responsibility attributed to others as well as high controllability and certainty of the situation. Thus, anger can lead to judgments or behaviors related to retaliation [24,25].

Anxiety, on the other hand, is linked to a generally low level of certainty and controllability about the situation. Thus, feeling anxiety can often induce behavior that can resolve the uncertainty (e.g., information seeking) [24,25]. Lastly, sadness is associated with the appraisal that the problem is under situational control (the belief that human cannot effectively control the situation) and the sense of irrevocable loss [24,25]. Therefore, sadness is often linked to passivity or withdrawal. Nabi [26] also proposed an emotions-as-frame model to explain the impact of media and emotion on attitudinal and behavioral consequences.

This model proposes that media messages trigger a range of discrete emotions based on the themes conveyed in those messages. For example, if the content of media suggests unfair offense to oneself, one is likely to feel anger about the given issue. Likewise, if the message implies uncertain negative outcome that can be a threat to oneself, the audience may experience anxiety or fear. Thus, according to this perspective, individuals can feel different emotions about important social issues or risks based on the unique themes conveyed in media messages.

Relatedly, research suggested that people feel different emotions depending on how they appraise risk or stress [24]. This, if people perceive a situation as threatening and out of control, stress emotions, such as anxiety, anger, and sadness-depression arise, thus, shaping their subsequent judgment and behavior. In fact, studies found that people feel more anger about environmental hazards, such as air pollution (compared to natural hazards) because they think that the problem is under human control [27]. Taken together, these theories and research illustrate that discrete emotions lead to distinct judgments and behaviors as these emotions are associated with unique cognitive appraisals about the situations.

Empirical research shows that the experience of discrete emotions indeed triggers distinct perceptions and judgments about risks. For instance, angry people show high certainty about the given situation and believe that they can control the situation, thus, becoming confident regarding their belief. As a result, angry people tend to underestimate the threats associated with a given risk. In support of this notion, studies found that anger leads people to be optimistic about risk [22]. In addition, as angry people are certain about the situation and are confident that they can fix the problem, they may engage in behaviors that can address the situation [28]. Indeed, it was found that anger promotes behavior that can resolve the problem associated with the risk [28].

On the other hand, anxious people tend to be less certain about the situation and are not confident about how to address the given problem. Therefore, this uncertainty about the situation leads people to perceive the risk as more threatening and devastating [29], as they become prepared to take measures that can resolve the potential negative outcomes. In support of this notion, it was shown that people who experienced anxiety were more motivated to participate in behavior that could reduce the risk than those in other emotions or control conditions [30].

In addition, people who feel sad or depressed about the situation were shown to perceive greater levels of risk because they believe that the problem was caused by external forces in which they had little control. Thus, feeling sadness leads people to be pessimistic about the possibility that they can control or resolve the risk situation, generating greater risk perception. In fact, it was found that sad individuals (those who watched a sad video) were more likely to interpret the given issue as more threatening than those in control conditions [31]. Furthermore, research examining the mechanism of coping or helping behavior showed that sadness can induce actions that change the current problematic situations [32] and motivate comfort-seeking behavior [30,33], thereby, leading individuals to take measures that can reduce a collective problem [34]. Thus, guided by findings from the existing literature, the following hypotheses are posed:

**Hypothesis** **1a** **(H1a).**  *Anger is negatively related to risk perception*.

**Hypothesis** **1b** **(H1b).**  *Anxiety is positively related to risk perception*.

**Hypothesis** **1c** **(H1c).**  *Sadness is positively related to risk perception*.

**Hypothesis** **2a** **(H2a).**  *Anger is positively related to policy support to address the risk*.

**Hypothesis** **2b** **(H2b).**  *Anxiety is positively related to policy support to address the risk*.

**Hypothesis** **2c** **(H2c).**  *Sadness is positively related to policy support to address the risk*.

### 2.2. SNS Dependency: Learning and Entertainment

The impact of SNS use on perceptions and judgments about risks may vary, depending on how individuals use SNS. Since individuals have different goals and purposes in using media [9], the ways people use SNS may lead to distinct consequences on risk judgments. In fact, specific types of SNS use were shown to be related to distinct judgments about risks. For instance, Jung et al. [35] examined the consumption of Zika-related news on social media and how it was related to perception of the risk of Zika virus infection.

They found that specific informational use, such as posting and viewing news regarding Zika on SNSs, was positively associated with preventive behavioral intention, while attention to Zika related news on SNSs moderated the relationship between posting news on SNSs and preventive behavioral intention. In the context of a MERS outbreak in South Korea, Oh et al. [7] found that exposure to MERS-related risk information on SNS positively influenced risk perception and preventive behavior.

Relatedly, Lee and Choi [36] investigated the relationship between the perceived credibility of false rumors and accuracy-oriented information seeking, particularly focusing on the role of SNS dependency during MERS outbreaks in South Korea. Their findings showed that while perceived credibility of false rumors did not influence accuracy-oriented information seeking, SNS informational dependency (versus SNS social dependency) interacted with false rumor credibility on accuracy-oriented information seeking. That is, the perception of false rumor credibility influenced information seeking behavior among those who relied on SNS for informational purposes, but not among those who relied on SNS for social purposes.

This suggests that using SNS to obtain information and increase understanding can motivate individuals to search for more accurate information about a specific risk issue. Taken together, these studies indicate that the effects of SNS use on perceptions and judgements about a risk are largely influenced by how individuals use SNS, such as the extent to which they use SNS to recognize, understand, and deal with important individual/social issues.

Extending existing literature on the impact of SNS use in risk-related judgments, the present study investigates how individuals’ general reliance on SNS to attain the goal of learning (versus entertainment) affects perceptions and judgments about risks. To do so, this study utilizes the concept of SNS dependency, which is rooted in the theory of media system dependency (MSD) [37,38]. MSD suggests the extent to which individuals rely on specific media or media genres is critical to decide the media’s impact. According to MSD, individuals’ overall dependency on certain media to achieve one’s goal, rather than the amount of time spent on the media or exposure to specific types of information, plays a more essential role in the impact of the media.

MSD identifies six types of goals individuals develop in using media—action orientation, interaction orientation, self-understanding, social-understanding, solitary play, and interactive play [38]. Among these goals, the present study focuses on the goals associated with learning, such as action orientation, interaction orientation, self-understanding, and social-understanding. For example, when individuals read news media articles about the introduction of vaccine, they may read it to obtain information about what they can do to get vaccinated (action orientation) or what they can do with their medical providers (interaction orientation).

They can also try to understand how being vaccinated may impact their health (self-understanding) and how it may affect public health and economy in general (social understanding). These goals can thus contribute to one’s learning and understanding about an issue. On the other hand, solitary and interactive play, such as skimming online contents, playing games, and chatting with others, may not increase one’s understanding of various issues as its main goal is to relax and have fun. In fact, even though play is considered an important aspect of the socialization process, where people can learn roles and values in society [38], entertainment-oriented activities on media have often been related to decreased interest in public affairs and civic issues [39].

Therefore, this study examines how a reliance on SNS for orientation and understanding (i.e., learning) is associated with people’s perceptions and behavioral judgments about the issue of fine dust pollution. Previous work documented that the impact of orientation and understanding dependencies intensify in uncertain and threatening situations [40] because, in times of risk, individuals want to rationalize what is happening, how the risk is relevant to themselves and others, and how to cope with the situation. Thus, individuals’ use of media in times of risk are likely to reflect these goals [41].

Specifically, the present study predicts that SNS dependency for learning is positively associated with perceptions of risk and policy support to address fine dust pollution. When individuals use SNS to obtain information about the actions that they can take (e.g., what they should do to minimize exposure to fine dust) and fathom the meaning of this risk on themselves and society in general (e.g., the possible health consequences on individuals and its effects on the country’s economy), this may naturally induce the perception that the risk can be a threat to themselves and lead them to support measures that can mitigate the risk. On the other hand, if individuals primarily use SNS for the purpose of entertainment, it may not influence the extent to which they understand about the issue of fine dust pollution, not impacting their perceptions or judgments about the risk. Thus, based on the insights gained from the literature, the following hypothesis is formulated:

**Hypothesis** **3a** **(H3a).**  *SNS learning dependency, not SNS entertainment dependency, is positively related to risk perception*.

**Hypothesis** **3b** **(H3b).**  *SNS learning dependency, not SNS entertainment dependency, is positively related to policy support*.

### 2.3. Moderating Role of SNS Dependency

The present study explores the potential moderating effects of SNS dependency on the relationship between emotion and judgments about risk. As discussed earlier, individuals’ perceptions and judgments about risks could vary according to the types of information obtained from media use. In particular, reliance on SNS to learn about various social issues may intensify the experience of relevant emotions regarding risk issues and amplifying the influence of these emotions on risk-related judgments. While watching/reading news on SNS or searching for political or financial information during crises, people may have increased opportunities to make cognitive appraisals of the risk issues (e.g., the threat or uncertainty of the issue and its potential negative consequences), thus, feeling discrete emotions associated with such appraisals.

Specifically, in the context of fine dust pollution in South Korea, SNS learning dependency is likely to increase people’s understanding about the nature of the fine dust problem, such as how threatening fine dust is to their health and the level of uncertainty regarding its short-term or long-term health consequences. These cognitive appraisals as the core theme of anxiety may strengthen the influence of anxiety on risk judgments [23,24,42].

Likewise, this type of SNS learning dependency might enhance the understanding regarding who is responsible for the fine dust pollution problem or what causes the risk (the core theme of anger) as well as the influence of the external or situational forces affecting the situation (the core theme of sadness/depression), which, as a result, may increase the influence of anger and sadness [23,24]. On the contrary, using SNS for entertainment may not interact with discrete emotions, since this type of use is not likely to influence cognitive appraisals of the situations relevant to the fine dust pollution. Therefore, the following hypotheses are posed:

**Hypothesis** **4a** **(H4a).**  *SNS learning dependency increases the influence of anger on risk perception*.

**Hypothesis** **4b** **(H4b).**  *SNS learning dependency increases the influence of anxiety on risk perception*.

**Hypothesis** **4c** **(H4c).**  *SNS learning dependency increases the influence of sadness on risk perception*.

**Hypothesis** **5a** **(H5a).**  *SNS learning dependency increases the influence of anger on policy support*.

**Hypothesis** **5b** **(H5b).**  *SNS learning dependency increases the influence of anxiety on policy support*.

**Hypothesis** **5c** **(H5c).**  *SNS learning dependency increases the influence of sadness on policy support*.

## 3. Method

### 3.1. Procedure and Participants 

To test our hypotheses and research questions, an online survey was conducted in Seoul between 20 February and 26 February 2019. Survey respondents were recruited from the online panel directory of a Seoul-based survey research firm in Korea. A cluster sampling procedure was used with three criteria: (1) gender, (2) age (20s, 30s, 40s, 50s, and older), and (3) the five main regions of South Korea. There were 6353 email invitations sent to potential respondents and 1560 respondents opened the email. A total of 958 respondents participated in the survey, and 510 respondents completed the survey (within-panel completion rate: 33%). About 49% of the respondents were female, and the mean age of respondents was 41.1 (SD = 12.6 years).

### 3.2. Measurement

#### 3.2.1. SNS Dependency

SNS dependency was measured by using questions developed in previous studies [35,42]. (Table 1). Respondents were first asked about the SNS service they use most often in their daily lives. Then, they answered how helpful they think the SNS (they chose) has been in their everyday lives in satisfying specific goals, on a five-point scale ranging from 1 (not helpful at all) to 5 (very helpful). Specifically, SNS learning dependency was measured by asking respondents how helpful the SNS has been in their everyday lives to understand how they should behave or interact with others in certain situations (i.e., action and interaction orientation) and to understand individual and social implications of certain events or issues (i.e., self and social understanding).

Likewise, SNS entertainment dependency was measured by asking the extent to which respondents us SNS to have fun and enjoy time with others (i.e., solitary and social play). A composite variable was created by calculating the mean score of the total item values for SNS learning dependency (Cronbach’s α = 0.88, *M* = 3.09, *SD* = 0.68) and for SNS entertainment dependency (Cronbach’s α = 0.80, *M* = 3.26, *SD* = 0.76).

#### 3.2.2. Risk Perception 

To measure risk perception, respondents were asked how threatening they think the fine dust problem is to people in general (M = 5.82, SD = 1.13) and to themselves (*M* = 5.95, *SD* = 1.12), using a 7-point scale ranging from 1 (not at all) to 7 (extremely). A risk perception index was created by using the mean score of general and personal risk perceptions (Cronbach’s α = 0.76, *M* = 5.89, *SD* = 1.01).

#### 3.2.3. Policy Support

Respondents were asked six questions to indicate their support for policies to mitigate fine dust pollution. Specifically, the questions asked if they support (1) providing incentives to companies that install facilities designed to reduce fine dust, (2) establishing a research fund for mitigating fine dust problem, (3) collaborating with foreign countries to reduce the fine dust pollution, (4) making provisions for fine dust issue, (5) penalizing companies and transportation systems that emit pollutants that cause the fine dust pollution, and (6) prosecuting business shutdown on companies that do not follow guidelines. Responses were measured on a 5-point scale ranging from 1 (not at all) to 5 (strongly support). Based on the mean score for each item, a policy support index was created (Cronbach’s α = 0.88, *M* = 4.03, *SD* = 0.62).

#### 3.2.4. Control Variables

Based on the literature that suggests sociodemographic factors influence risk perception and judgment, this study included age (*M* = 41.1, *SD* = 12.6), gender (male = 51.2%), educational achievement (high school or less: 24.9%; college or bachelor’s degree: 64.5%; more than bachelor’s degree: 10.6%), and political orientation (ranging from 1–5, a higher score indicating more conservative; *M* = 3.65, *SD* = 1.12) as control variables in the analyses. In addition, general media use, such as traditional media use (e.g., television, radio, and newspaper) (*M* = 2.65, *SD* = 1.50), internet use (*M* = 5.51, *SD* = 2.01), and social media use (*M* = 3.23, *SD* = 2.31) were controlled in the analyses (ranging from 1–7, a higher score indicating more time spent on the media).

### 3.3. Analysis Strategy

To examine the hypotheses of this study, two sets of hierarchical regression analyses were conducted, one for risk perception and another for policy support, using STATA 14.2. Sociodemographic factors (age, gender, education, and political orientation) and general media use variables (traditional media, Internet, and social media) were entered in the first block. Next, discrete emotions (anxiety, anger, and sadness) and SNS dependency (learning and entertainment) were entered in the second block. Lastly, the interaction terms between emotions and SNS learning dependency were created and entered in the third block. Discrete emotions and SNS dependency variables were mean-centered to reduce multi-collinearity in the interactive regression model [43].

## 4. Results

Before testing the hypotheses, correlational analyses between the main variables of this study were conducted for preliminary analyses purposes (Table 2). The partial correlational analyses indicate some significant correlations between sociodemographic factors and risk perception and policy support. Educational attainment was positively correlated with risk perception (*r =* 0.09, *p <* 0.05) and sociodemographic factors were significant correlated to policy support. Specifically, older (*r =* 0.18, *p <* 0.001), female (*r =* 0.10, *p <* 0.05), highly educated (*r =* 0.12, *p <* 0.01), and being conservative (*r =* −0.15, *p <* 0.001) were positively and significantly related to support for policy.

With regard to general media use, the use of traditional media (*r =* 0.10, *p <* 0.05) and Internet use (*r =* 0.28, *p <* 0.001) were positively related to risk perception. In contrast, the use of social media was negatively related to risk perception (*r =* −0.14, *p <* 0.01). Similarly, traditional media (*r =* 0.23, *p <* 0.001) and Internet use (*r =* 0.16, *p <* 0.001) were positively related to policy support while social media use was negatively related to support for policy (*r =* −0.10, *p <* 0.05).

### 4.1. Risk Perception

As shown in Table 3, the results of hierarchical regression predicting risk perception indicated that some sociodemographic factors and media use variables influence risk perception regarding the fine dust pollution. Being conservative was negatively associated with risk judgment (*β* = −0.6, *p <* 0.05). On the other hand, using the internet was positively associated with risk perception (*β* = 0.04, *p <* 0.05). The multiple regression analyses revealed that discrete emotions as a whole explain 43% of the variance in risk perception. H1a–c predicted that anger would be negatively, and anxiety and sadness would be positively related to risk perception.

The results indicate that anxiety (*β* = 0.35, SE = 0.04, *p <* 0.001) and sadness (*β* = 0.06, SE = 0.03, *p <* 0.01) about the fine dust issue were significantly and positively associated with the perception of risk, supporting H1a and H1c. However, different from our prediction that anger might be negatively related to risk perception, feeling anger about the issue was positively related to risk perception (*β* = 0.17, SE = 0.04, *p <* 0.001). Thus, H1b was not supported. In addition, this study assessed whether reliance on SNS for learning is positively related to the perception of risk. The results indicated that SNS dependency for learning is not associated with risk perception (*β* = 0.02, SE = 0.06 *p = n.s.*), thus, rejecting H3a.

### 4.2. Policy Support

Table 4 presents the results of hierarchical regression predicting policy support. Again, the results showed that some control variables influence support for policy to address the fine dust risk. Specifically, age was positively related to policy support (*β* = 0.08, SE = 0.02, *p <* 0.001). Being conservative was negatively associated with support for policy (*β* = −0.06, *p <* 0.01), with conservatives less likely and liberals more likely to support fine dust air pollution mitigation policies. This result seems to make sense, considering the fact that the current ruling party is the Democratic Party. Among the media use variables, traditional media use (*β* = 0.05, SE = 0.02, *p <* 0.05) was positively and social media use *(β* = −0.19 SE = 0.06, *p <* 0.01) was negatively related to policy support. Again, multiple regression analyses indicated that discrete emotions as a whole explain 24% of the variance in public support for policies to address the fine dust air pollution.

In H2a-c, this study expected that feelings of anxiety, anger, and sadness would be positively associated with policy support. The results show that feeling anxiety (*β* = 0.18, SE = 0.04, *p <* 0.001), anger (*β* = 0.08, SE = 0.04, *p <* 0.05), and sadness (*β* = 0.08, SE = 0.03, *p <* 0.01) about the issue were all significantly and positively related to support for policy, lending support for H2a-c. In addition, the results showed that SNS dependency for learning was positively associated with policy support (*β* = 0.11, SE = 0.06, *p =* 0.06). Thus, H3b was supported.

### 4.3. Interaction Effects

This study also examined the interaction between discrete emotions and SNS dependency for risk perception (H4a–c) and policy support (H5a–c). With respect to H4a–c, regarding the potential interaction between discrete emotions and SNS learning dependency for risk perception, this study predicted that the impact of feeling discrete emotions—i.e., anxiety, anger, and sadness—on risk perception might increase when individuals are highly dependent on SNS for learning. The results confirm an interaction effect between SNS dependency for learning and anxiety about the issue for risk perception.

Specifically, the effects of anxiety on risk perception increased when respondents were highly dependent on SNS for learning (*β* = 0.24, SE = 0.10, *p <* 0.05), lending supporting for H4a. However, such interaction effects were shown neither for anger (*β* = 0.17, SE = 0.11 *p =* 0.13) nor for sadness (*β* = −0.10, SE = 0.08, *p =* 0.24). Thus, H4b–c were not supported. Additionally, the results showed that SNS entertainment dependency negatively interacted with feeling anxiety, suggesting that the effects of anxiety on risk perception decreased when respondents were highly dependent on SNS for entertainment (*β* = −0.18, SE = 0.08, *p <* 0.05).

**Figure 1:**
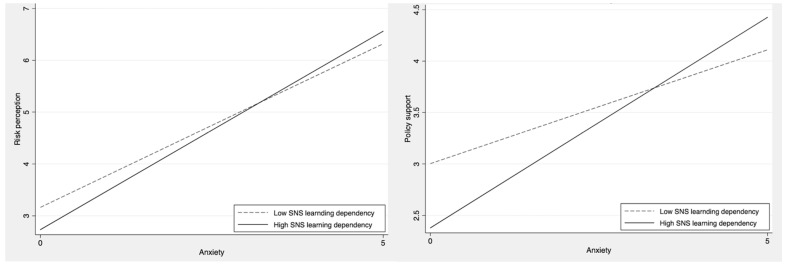
The effects of anxiety on risk perception (on the **left**) and policy support (on the **right**) moderated by SNS learning dependency.

Regarding H5a–c, the present study assessed whether the impacts of anxiety, anger, and sadness on policy support might increase among those who are highly dependent on SNS for learning. The results again revealed that only anxiety interacts with SNS learning dependency: the impact of anxiety on policy support increased when individuals were highly dependent on SNS for learning (*β* = 0.22, SE = 0.10, *p <* 0.05), lending supporting for H5a. Such interaction effects were shown neither for anger (*β* = −0.01, SE = 0.08 *p =* 0.88) nor for sadness (*β* = −0.04, SE = 0.06 *p* = 0.53), thus, rejecting H5b,c. Figure 1 illustrates the interactions between anxiety and SNS learning dependency on risk perception and policy support.

The positive impact of anxiety on risk perception was stronger for those with higher SNS learning dependency compared to those with lower SNS learning dependency (Figure 1, on the left). Likewise, the role of anxiety in policy support was greater for those with higher SNS learning dependency compared to those with lower SNS learning dependency (Figure 1, on the right). Taken together, these results suggest that the impact of anxiety on risk perception and policy support could significantly increase when individuals use SNS for the purpose of learning.

## 5. Discussion

This study explored how individuals’ discrete emotions felt toward a public risk issue (i.e., fine dust pollution) guide their perceptions of risk and behavioral judgments. In particular, considering that social media have become a prominent communication platform in the context of risk, this study examined how individuals’ reliance on SNS influences their risk-related judgments and how, if at all, it might moderate the relationship between discrete emotions, perceived risk, and policy support. Overall, the findings suggest that discrete emotions are indeed distinctively associated with perceptions of risk and support for policies to mitigate the risk. Furthermore, SNS dependency for learning has a significant and positive influence on policy support, while simultaneously intensifying the effects of anxiety on risk perception and policy support.

First, the findings indicate that feeling anxiety, anger, and sadness about the fine dust risk issue are all positively related to perceptions of risk. In line with previous research that suggests that anxiety states are associated with the tendency to favor more threatening interpretation of ambiguous situations [44], respondents who felt a high level of anxiety estimated the fine dust pollution as more threatening. Respondents might have felt that they had no control over the situation [45], thus, experiencing a greater level of risk perception. Sadness was also positively linked to risk perception, consistent with previous work that sadness tends to decrease the unrealistic illusion of control [3].

Indeed, as sadness signals helplessness about a loss [24], it might have led to increased risk perception. On the other hand, different from our prediction that anger might be negatively related to risk perception, anger was also positively related to perceived risk. One possibility is that angry individuals were more motivated to blame others who seem to be responsible for the risk [24], thus, perceiving the problem as more serious. That is, the perception that other people unfairly and negatively affect their lives might have led individuals to assess the risk as worse. In line with this reasoning, previous work found that attribution of responsibility to other(s) tend to generate negative perceptions about the general states of the problem [46].

The findings also suggest that anxiety, anger, and sadness are all positively related to policy support. Consistent with prior work that showed anxiety increases risk-preventive behavior [30] and anger induces the motivation to address the problem to eliminate the potential barrier of goals [23,24], anxiety and anger motivated people to support measures that can reduce the risk [47]. In addition, sadness was linked to increased policy support. Despite some research that suggests sadness instigates an inaction tendency, the findings of our study indicate that sad individuals were more likely to act on the problem. It is possible that sadness, by leading individuals to slow down and pay attention to details [48], might have enhanced careful processing of risk-related messages, thus, leading to increased engagement to address the risk. Taken together, the findings point out that discrete negative emotions indeed have the potential to alert people to risks and social problems and to engage with the risks.

With respect to the influence of SNS dependency on perceived risk and policy support, the findings indicate that using SNS to obtain information and increase one’s understanding about various individual and social issues (i.e., SNS learning dependency) is positively related to policy support to mitigate the risk, while this effect was not present for risk perception. First, the finding that SNS learning dependency was not associated with risk perception appears to indicate that SNS dependency itself does not exert a direct influence on specific perceptions about the risk.

While previous research found that the informational use of SNS was positively related to increased risk perceptions about the given risks [44], these studies primarily assessed the use of SNS by measuring how individuals use specific information, which was directly related to the issue, on the media. However, since a general reliance on SNS for learning is not directly related to increased knowledge or understanding about the specific issue at hand, SNS dependency might not be able to directly influence specific perceptions about the risk, such as how threatening a certain issue might be.

On the contrary, the findings demonstrate that the respondents with high SNS learning dependency were more likely to support measures for addressing the risk. It is possible that individuals who are dependent on SNS for learning, while increasing their general knowledge and understanding about important social issues and events surrounding them, might have been more motivated to engage in a range of social actions that can reduce public risk. That is, even though they might have not increased specific knowledge related to specific issues, heightened interest in public life and social issues might have led them to make more civic judgments.

This finding is particularly promising considering that previous research that found a positive link between SNS use and policy support primarily focused on how exposure to information about a specific risk issue promotes public engagement with the risk [7,49]. Our findings further suggest that alongside the direct search for relevant information on social media, a general reliance on SNS for learning in everyday lives also has beneficial effects on boosting public engagement with social problems. On the other hand, relying on SNS for entertainment was not significantly associated with risk perception and policy support. It seems that respondents who primarily relied on SNS for resting or entertainment might have had relatively little chance to learn and understand various social issues. As a result, they were not alerted by social risks, resulting in less interest or motivation to engage in actions that could reduce public risk.

Another interesting finding of this study is the moderating effects of SNS dependency on the relationship between discrete emotions, risk perception, and policy support. Specifically, when the respondents were highly dependent on SNS for learning, feeling anxious about the risk issue had a greater impact on the perceptions of risk as well as the intention to support policies for addressing the risk. It can be inferred from the findings that individuals who use SNS to increase their understanding about various individual or societal issues are more motivated to feel the issue, as they have more chances to make cognitive appraisals of the issue, such as the extent to which the issue is controllable or how much uncertainty the issue involves.

Specifically, these individuals might have frequently encountered information on SNS that can be related to appraisal themes of anxiety rather than those related to sadness or anger. Consequently, this type of SNS dependency might have strengthened the effects of anxiety on risk perception and behavioral judgments. Since this is only one possibility among others, future studies that examine the actual content or information on SNS may elucidate the findings obtained in this study.

This study offers important theoretical and practical implications about the role of emotions in public health crises. Our study shows that the respondents who were worried about fine dust pollution were the most likely to perceive a greater risk and engage in measures to resolve the risk compared to those who were angry or sad. In line with previous research that suggested that anxiety plays a crucial role in engaging individuals with risks [50], the findings of this study provide additional evidence that anxiety indeed exerts a powerful influence on shaping public perceptions and behaviors. Accordingly, professionals and campaign practitioners could develop communication strategies that are designed to induce the feeling of anxiety to maximize the possibility that individuals are motivated to engage with important risk issues.

Furthermore, the findings suggest that research on media effects and risk communication can provide a more nuanced explanation of when and how discrete emotions shape judgment and behavior in specific ways. Our findings indicate that this could be done by considering the role of specific types of SNS usage in the process. Specifically, the findings illustrate that anxiety is a particularly vital influencer of the public’s risk perception when individuals use SNS for learning. This shows that the influence of emotions can be better understood by considering the complex interplay of discrete emotions, distinct patterns of SNS use, and judgments about risks.

Despite the significance of the findings obtained in this study, it is important to note that this study has several limitations. First, this study focused on three specific negative emotions: anxiety, anger, and sadness. While this was due to the consideration that these emotions are the critical emotions that have been shown to affect subsequent judgement and behavior in various risk contexts, examining the influence of other emotions—for example positive emotions—may extend the research in this field. For instance, previous research showed that positive emotions, such as hope, positively influenced people’s engagement with the risk issue [51].

Future research that examines how specific positive and negative emotions can influence aspects of risk-related judgments could unearth further insights regarding the role of emotions in judgments about risk. In addition, the present study examined the effects of SNS dependency by asking the respondents to choose one SNS that they used the most and their reliance on the media. Even though this has been widely used in prior work on social media dependency [36], future research that employs different measures of SNS dependency will help clarify the findings obtained in this study.

For example, examining the potentially differential impact of reliance on video or text-oriented SNS or private or public SNS could be fruitful. Moreover, while previous studies suggested that certain individual characteristics, such as self-efficacy [52], knowledge [53], or personal experience [54], play a crucial role in perceptions and judgments about risks, the present study did not examine the potential impact of these variables. Studies that further examine the role of these individual characteristics will help advance our understanding about the relationship between emotions, social media use, and risk judgments.

Perhaps more importantly, the unique nature of the fine dust pollution issue employed in the current study warrants special attention. As discussed earlier, the issue of fine dust pollution in South Korea has been considered a problem that contains high uncertainty and low controllability. In addition, it is important to note that, compared to other more personal health-related risks, the issue of air pollution is considered to have less immediate effects on individuals’ health. For example, when the risk is more directly related to individual health and contains high certainty, such as in the case of skin cancer [31], the effects of discrete emotions might be shown differentially since this type of risk can bring about more immediate and direct fatal consequences on health.

Furthermore, the fine dust problem has often been believed to originate from other countries, such as China [14]. This points toward the possibility that the findings obtained in this study might be shown differentially for other types of issues, especially for those with different attributions of responsibility. Therefore, the findings obtained in this study should be understood in relation to this specific type of risk and context. Future research could further examine the potentially distinct effects of discrete emotions or SNS use for different types of risks.

In addition, the fact that this study is based on cross-sectional data raises concerns about the causal relationships. Despite the fact that the present study assumed that discrete emotions and SNS use may influence risk perception and policy support based on theories and research findings, the inverse relationship is also plausible. For instance, one could argue that individuals who perceive greater risk could feel a greater level of anxiety about the risk issue [55]. Thus, future studies could elucidate the causal relationship suggested in this study using different study designs, such as experimental or panel designs.

## 6. Conclusions

This study aimed to extend previous research on the effects of discrete emotions on public responses to risk by examining how discrete emotions, coupled with SNS use, shape public risk perception and judgment. The findings illustrate that both discrete emotions and SNS use can be vital shapers of public risk perception and that the impact of emotions can be amplified by using SNS for specific purposes (i.e., learning). Future research that addresses the aforementioned limitations of this study will advance our understanding about the relationship between emotion, social media use, and public response to risk.

For example, studies could examine a broader range of relevant emotions while considering the impact of situational or individual factors in the process. Indeed, future research could further probe the intricate interplay of discrete emotions and social media use across different types of risk in different contexts. This will help provide a clearer picture of how discrete emotions influence the public’s risk perception and motivation to engage with the risk.

## Figures and Tables

**Table 1:** Questions used for measuring SNS dependency.

Heading	Goals	Questions	*M* (*SD*)	
	Action orientation	To decide where to get particular services To get information on purchasing goods When I want to plan an evening or weekend of leisure time	2.82 (1.07)	
			3.17 (1.01)	
			3.12 (0.97)	
SNS learning dependency	Interaction orientation	To know how to interact with other people To know how to react to others To learn how to interact with a counterpart at an important meeting	3.55 (0.96)	
			3.50 (1.01)	
			3.07 (0.99)	3.09 (0.68) α = 0.88
	Self-understanding	To look back on my behaviors To think about my future life plans To know how others reacted when they were in situations similar to mine	2.90 (1.03)	
			2.68 (1.07)	
			3.34 (0.98)	
	Social understanding	To know what is going on in the world To know the major current issues in my country To know what is going on in other countries	3.16 (1.02)	
			3.04 (1.08)	
			3.00 (1.10)	
SNS entertainment dependency	Solitary play	To rest after a long day To have quiet time on my own To find things to do when I am alone	3.25 (1.10)	
			2.82 (1.24)	
			3.20 (1.11)	3.26 (0.76) α = 0.80
	Social play	To find things to do with my friends To have fun with my family/friends To enjoy an event without being there	3.62 (1.03)	
			3.52 (1.03)	
			3.16 (1.03)	

**Table 2:** Point biserial correlations among the variables of this study.

	1.	2.	3.	4.	5.	6.	7.	8.	9.	10.	11.	12.	13.
1.													
2.	0.01												
3.	0.07	−0.10 *											
4.	−0.02	−0.05	−0.09 *										
5.	0.22 ***	0.07	0.07	−0.01									
6.	−0.04	0.11 *	0.11 *	−0.05	0.25 ***								
7.	−0.07	−0.11 *	−0.01	−0.05	0.36 ***	0.39 ***							
8.	−0.05	0.07	0.09	0.01	0.17 ***	0.27 ***	0.22 ***						
9.	−0.03	0.16 **	0.08	−0.04	0.14 **	0.33 ***	0.25 ***	0.65 ***					
10.	0.01	0.14 **	0.13 **	0.01	0.18 ***	0.13 **	0.13 **	0.64 ***	0.50 ***				
11.	0.10 *	0.02	−0.01	0.04	0.19 ***	0.05	0.15 **	0.06	0.02	0.06			
12.	−0.04	0.02	−0.05	0.06	0.11 *	0.06	0.13 **	0.08	0.02	0.02	0.79 ***		
13.	0.03	0.03	0.09 *	−0.08	0.10 *	0.28 ***	0.14 **	0.54 ***	0.64 ***	0.40 ***	0.08 *	0.12 **	
14.	0.18 ***	0.10 *	0.12 **	−0.15 ***	0.23 ***	0.16 ***	0.10 *	0.42 ***	0.45 ***	0.39 ***	0.15 ***	0.10 *	0.54 ***

*Note.* 1. Age; 2. Gender; 3. Education; 4. Partisanship; 5. Traditional media; 6. Internet; 7. Social media; 8. Anger; 9. Anxiety; 10. Sadness; 11. SNS learning dependency; 12. SNS entertainment dependency; 13. Risk perception; and 14. Policy support. *Note.* * *p* < 0.05. ** *p* < 0.01. *** *p* < 0.001.

**Table 3:** Hierarchical regression analyses predicting risk perception.

Variables	Risk Perception		
	Step 1	Step 2	Step 3
	*β*	*β*	*β*
Sociodemographic factors			
Age	−0.05 (0.03)	−0.02 (0.02)	−0.02 (0.02)
Gender	0.03 (0.06)	−0.08 (0.05)	−0.08 (0.05)
Education	0.05 (0.03)	0.02 (0.03)	0.02 (0.03)
Political orientation	−0.04 (0.03)	−0.04 + (0.02)	−0.04 + (0.02)
Media use			
Traditional media	0.01 (0.02)	−0.02 (0.02)	−0.02 (0.02)
Internet	0.08 *** (0.02)	0.03 * (0.01)	0.02 + (0.01)
Social media	0.03 * (0.02)	0.01 (0.01)	0.01 (0.01)
Negative emotions			
Anxiety		0.35 *** (0.04)	0.20 (0.22)
Anger		0.17 *** (0.04)	0.20 (0.20)
Sadness		0.06 * (0.03)	0.06 (0.13)
SNS learning		0.02 (0.06)	−0.55 (0.34)
SNS entertainment		0.02 (0.05)	0.40 (0.30)
Anxiety * SNS learning			0.24 * (0.10)
Anxiety * SNS entertainment			−0.18 * (0.08)
*R* ^2^	0.11	0.48	0.50
Adjusted *R*^2^	0.10	0.47	0.48
Adjusted *R*^2^ change		0.37	0.01
*F*	7.99 ***	33.60 ***	24.64 ***

*Note.* In step 3, only significant interactions were included in the regression model. *Note.* * *p* < 0.05. *** *p* < 0.001.

**Table 4:** Hierarchical regression analyses predicting policy support.

Variables	Policy Support		
	Step 1	Step 2	Step 3
	*β*	*β*	*β*
Sociodemographic factors			
Age	0.06 * (0.02)	0.07 ** (0.02)	0.07 ** (0.02)
Gender	0.13 * (0.05)	0.06 (0.05)	0.05 (0.05)
Education	0.06 * (0.03)	0.03 (0.02)	0.02 (0.02)
Political orientation	−0.07 ** (0.02)	−0.08 *** (0.02)	−0.09 *** (0.02)
Media use			
Traditional media	0.07 ** (0.02)	0.05 * (0.02)	0.05 * (0.02)
Internet	0.03 * (0.01)	0.01 (0.01)	0.01 (0.01)
Social media	−0.00 (0.01)	−0.01 (0.01)	−0.19 ** (0.06)
Negative emotions			
Anxiety		0.18 *** (0.04)	−0.29 (0.21)
Anger		0.08 * (0.04)	0.10 (0.19)
Sadness		0.08 ** (0.03)	0.19 (0.13)
SNS learning		0.11+ (0.06)	−0.50 (0.34)
SNS entertainment		0.02 (0.05)	0.30 (0.29)
Anxiety * SNS learning			0.22 * (0.09)
Anxiety * SNS entertainment			−0.07 (0.08)
*R^2^*	0.12	0.34	0.37
Adjusted *R^2^*	0.11	0.32	0.34
Adjusted *R^2^* change		0.21	0.02
*F*	8.62 ***	18.31 ***	16.46 ***

Note. * *p* < 0.05. ** *p* < 0.01. *** *p* < 0.001.

## Data Availability

Data are available on request due to ethical issues.
